# Forensic uses of research biobanks: should donors be informed?

**DOI:** 10.1007/s11019-015-9667-0

**Published:** 2015-09-29

**Authors:** Vilius Dranseika, Jan Piasecki, Marcin Waligora

**Affiliations:** REMEDY, Research Ethics in Medicine Study Group, Department of Philosophy and Bioethics, Jagiellonian University Medical College, Michalowskiego 12, 31-126 Krakow, Poland; Department of Logic and History of Philosophy, Vilnius University, Vilnius, Lithuania

**Keywords:** Decision-making, Donation/procurement of organs/tissues, Informed consent, Human tissue, Government/criminal justice

## Abstract

Occasional reports in the literature suggest that biological samples collected and stored for scientific research are sometimes accessed and used for a variety of forensic purposes. However, donors are almost never informed about this possibility. In this paper we argue that the possibility of forensic access may constitute a relevant consideration at least to some potential research subjects in deciding whether to participate in research. We make the suggestion that if some type of forensic access to research collections is likely to be perceived by the subjects as a reason against donating their biological materials, there are good ethical reasons to make this type of access impossible or at least severely restricted. We also provide an ethical argument for the claim that, if a total ban on this type of forensic access cannot be achieved, potential research subjects should be informed about the extent to which this type of forensic access is possible.

## Background

The past several decades have seen a rapid expansion of biobanks created specifically for the purposes of forensic investigation, and DNA evidence is increasingly often accepted in courts (Lee, Crouse and Kline 2013; Butler 2009). For example, the number of offender profiles in the US National DNA Index grew fourfold between 2005 and 2015, from less than 3 million to almost 12 million (Butler 2009, p. 269; The Federal Bureau of Investigation 2015). The US National Institute of Justice indicates that “state and local [forensic] DNA laboratories increase[d] capacity almost fourfold between 2005 and 2010. These capacity improvements in the nation’s DNA laboratories have allowed DNA laboratories to keep pace with the demand for new DNA services, which has also increased almost fourfold” (National Institute of Justice 2012). Similarly, in its 2007 report on ethical issues in forensic use of bioinformation, the Nuffield Council on Bioethics refers to a “dramatic increase in the forensic use of bioinformation” (p. xiii).

However, it is not only these specialist biobanks that are used for forensic purposes. Occasional reports in the literature indicate that biological samples collected for scientific research, medical diagnostics and screening, and other non-forensic purposes are sometimes used in different countries—including Australia, New Zealand, Norway, the UK, and Sweden—for a variety of forensic purposes, such as criminal identification, disaster victim identification or paternity identification (Bowman and Studdert 2011; McCartney 2004; Kaye 2006; Hansson and Bjorkman 2006). One of the first widely publicized cases was that of Stephen Kelly, who was convicted in Scotland for recklessly passing on the HIV virus through sexual intercourse. The scientific evidence that led to the conviction was derived from an earlier biological sample, obtained from the researchers by a police warrant (Dyer 2001). Further widely publicized examples of forensic access to non-forensic collections were the cases of access to blood samples from the PKU biobank in Sweden for investigation of the murder of Swedish foreign minister Anna Lindh in 2003 and identification of victims of the 2004 Indian Ocean tsunami (Hansson and Bjorkman 2006).

Information on forensic access to non-forensic biobanks is limited to reports on individual cases, and there is no systematic information available on the frequency and types of forensic access. Neither is it known whether forensic access to non-forensic collections is becoming more prevalent over time. Some commentators, however, warn of a “function creep,” whereby police departments or other agencies push to gain easier access to medical research DNA databases for forensic purposes (McCartney 2004, p. 163; Kaye 2006, p. 16). In order to assess this warning it would be very helpful to know how often forensic access to non-forensic collections is attempted (and what types of it), and how frequently it is granted, as well as to what extent current legal provisions in fact preclude types of forensic access that may be found objectionable by potential donors of biological materials. Unfortunately, no systematic evidence is available to address these issues.

Our discussion in this article focuses on forensic use of research collections, but much of what is said could be extended to other types of non-forensic collections, storing samples collected for medical diagnostics or screening. Furthermore, the distinction between research collections and diagnostics/screening collections is sometimes blurred. For example, blood samples in PKU biobanks are collected for screening purposes, but one of the reasons—and perhaps the main reason—for long-term storage of these samples is their potential for research.

In what follows, we briefly examine two types of tension between the different interests that arise in the context of forensic access to non-forensic biobanks. We argue that these two tensions result in a dilemma: it seems difficult to secure both donors’ willingness to donate their biological materials and their being informed of all relevant aspects of the study. We discuss three ways to navigate this dilemma and argue that if a given type of forensic access to research collections is likely to be perceived by the subjects as a reason against donating their biological materials, there are good ethical reasons to make this type of access impossible, or at least severely restricted. We also provide an ethical argument for the claim that, if a total ban on this type of forensic access cannot be achieved (and, in many countries, the possibility of a total ban seems very unlikely), potential research subjects should be informed about the extent to which this type of forensic access is possible.

## Two types of tension and a dilemma

Forensic access to identifiable biological materials and information stored in research biobanks (herein called forensic access) creates at least two types of tension between different interests (Hofmann 2006, p. 129; Seiden and Morin 2002, p. 92). The first tension arises between (1) societal interest in public trust in research biobanks and medicine more generally and (2) societal interest in law enforcement. The wide availability of information on forensic access may result in diminished trust in research biobanks and diminished willingness to donate biological materials for research. The second tension arises between (1) individual interests of donors in privacy, confidentiality and control of one’s own biological samples and health data and, again, (2) societal interest in law enforcement. Forensic access involves privacy and confidentiality risks, and if information on forensic access is unavailable this may result in lack of control over what happens to one’s own biological samples and health data. When combined, these two tensions result in a dilemma: if donors are informed about potential forensic use, they may be reluctant to donate their materials to research; and if donors are not informed about potential forensic use, they cannot use this information in deciding whether research participation is in their best interest and whether the resulting privacy and confidentiality risks are acceptable to them. Let us briefly describe the two horns of this dilemma.

## Can forensic access make a difference to donors’ willingness to donate their materials?

The claim to the effect that forensic access could undermine public trust and discourage research participation is often encountered in the literature (Hansson and Bjorkman 2006, p. 285; Tamburrini 2011, p. 137; Bexelius et al. 2007, p. 442; Cho and Sankar 2004). If informed about forensic access, some subjects might be concerned that the collected data could be used against their interests, consider forensic access to be an infringement of their privacy, or perceive the possibility of forensic access as a sign of the encroaching power of the state over individuals. Such motives, the argument goes, can lead to a situation in which it becomes more difficult to collect and retain biological samples, especially when samples are intended for long-term storage.

Data on public attitudes toward forensic access offer conflicting accounts. For example, a study by David Kaufman and his colleagues found that 84 % of 5000 adult research subjects in the USA “felt that it would be important to have a law protecting research information from law-enforcement officials,” 75 % were concerned about the government having their samples and information, and 37 % were afraid that research data could be used against them (Kaufman et al. 2009). At the other extreme, in a study conducted in Sweden in 2005 “a majority (88.1 %) of the respondents thought that it would be acceptable for the police to gain access to genetic samples stored in relation to healthcare,” and only 6.3 % indicated that this “would have a negative impact on their trust in the healthcare services” (Bexelius et al. 2007, p. 442). It is difficult to tell to what extent these extreme differences are due to variations in study methodology, different political traditions in the two countries, or even perhaps the timing of the study—the Swedish survey was conducted shortly after forensic access to databanks led to arrests in relation to the murder of Anna Lindh and the identification of victims of the tsunami.

Despite these differences, it seems safe to say that at least in some countries a significant portion of the population disapproves of at least *some types* of forensic access to research biobanks. Therefore, information on such forensic access can compromise the trust of at least this part of the population. More fine-grained survey methods are needed to establish whether different types of forensic access are perceived by the public to be objectionable to different degrees.

The negative attitude of at least a portion of the public to forensic access gives a reason to expect that for some people, if they were alerted to the fact that such access occurs, this information could be important in their own choices as to whether to donate their materials. For example, the UK Biobank Ethics and Governance Council reports a study in which some respondents of the UK Biobank survey were “concerned about potential miscarriages of justice arising from technical mistakes and the planting of genetic evidence” if biological materials were routinely made available to the police (Webster et al. 2008; see also Kaufman et al. 2009; Lewis et al. 2013, p. 8). Others may have more general qualms over the power of the state over the citizens and thus be unwilling to participate.

Some behavioral evidence is also available that suggests that people sometimes request the destruction of their samples. For example, Claudio Tamburrini reports that approximately 2000 people requested the destruction of their previously donated PKU samples after forensic use of biobanks was covered by the media in Sweden in 2003 (2011, p. 137). The efforts invested in such requests suggest that at least some people think that the possibility of forensic access is perceived by them to be against their interests. It is not possible, however, to determine whether such requests are driven by concerns over forensic applications, or other, unrelated privacy considerations.

Similar concerns can be raised over collections that were collected for diagnostics or screening. It would be interesting to ascertain the influence of availability of information on potential forensic access on people’s willingness to allow their samples to be stored in a medical setting for such purposes as re-diagnosis or quality assurance.

## Is forensic access a relevant consideration?

If we agree that information on forensic access can have a negative influence on donors’ willingness to donate their materials, this brings us to the second horn of the dilemma: should donors’ participation be secured by not informing them about the potential forensic use? There is a long-standing debate on the amount and content of information that should be provided to research participants. Discussions on consent in biobanking mostly concern the scope of possible future research. However, subjects may be interested in issues that are not directly related to research, but rather to research infrastructure and safety measures, such as how confidentiality will be assured and how third-party access will be organized. Such considerations may well influence donors’ decisions as to whether to donate their materials. And if decisions can depend on such a consideration, it is arguably in the interest of a participant to have this information and be able to incorporate it into her decision process. As expressed by Bromwich and Millum, “When the researcher withholds information about a risk that she reasonably believes would be relevant to the prospective participant’s enrolment decision, she arrogates his role as agent by determining what information he gets to consider” (Bromwich and Millum 2013, p. 8; see also Feinberg 1984, p. 307). Are there reasons to think that information on potential forensic access constitutes such a relevant consideration? The data summarized in the previous section give ample reason to believe that at least for a portion of the public some forensic uses may be such relevant considerations. And if a researcher knows that, withholding this information from potential donors constitutes wrongful deception.

Arguably, the various types of forensic access and risks associated with such uses are likely to be perceived differently, and it may therefore be unhelpful to speak about forensic access indiscriminately. For example, it may be the case that people’s attitudes toward forensic access for criminal identification are significantly more negative than their attitudes towards forensic access for disaster victim identification. Consequently, it may well be possible that only some types of forensic access will constitute relevant considerations. Furthermore, it may be the case that targeted access to particular samples—for instance to confirm someone’s identity—may be perceived as more acceptable than full-scale database searches. Such considerations can justify different regimes of protection applied to different types of forensic access.

The possible variability of attitudes toward different types of forensic access seems to be a fertile ground for research with potential policy implications. In what follows we will use the phrase “relevant types of forensic access” to signify “those types of forensic access that are likely to be relevant considerations in a given society.” Which types of forensic access count as relevant is, to a large extent, an empirical question.

Having discussed the dilemma, we now proceed to three ways to navigate it.

## The first solution: ignore it

Forensic access is something that is salient to very few potential donors of biological materials. The existence of such access does not seem to be part of general knowledge. Furthermore, currently research participation consent forms, including consent to donate materials to biobanks, do not mention this possibility (with very few exceptions, which will be addressed later in the article). So one may say: let it stay that way! A number of reasons can be mentioned to justify this option: information on forensic use is not directly connected with the primary purpose of a research biobank; forensic use is a very remote possibility; consent forms are already too long.

This strategy may seem to have an important advantage—donors, if unaware of potential forensic access of relevant types, will be less likely to refuse to donate their materials. Trust, however, can be eroded if information on such access becomes available. This is especially likely in countries with low trust in government and public institutions. Another problem with this strategy is that it may constitute wrongful deception of donors by researchers—knowingly suppressing information that is likely to be relevant for the choice of whether to participate. The remoteness of the possibility does not necessarily make it irrelevant to the donor’s choice. Furthermore, the possibility of forensic access can be expressed in a short and simple sentence, and will therefore not add much to the complexity of consent forms—especially bearing in mind the fact that consent forms routinely contain pieces of information that are much less likely to be relevant considerations. These arguments provide a rather strong case against this strategy. So let us discuss the alternatives.

## The second solution: ban it

Another option is making relevant types of forensic access to research collections legally impossible. This has at least two benefits. First, by clearly separating forensic collections from research collections it removes the potential of forensic access to decrease public trust in and support for research and, consequently, participation. Second, if relevant types of forensic access are made impossible, the issue of deception by withholding from donors relevant information no longer arises. In fact, this option obviates the need for disclosure.

This option is attractive, and has been defended repeatedly in the literature (Tamburrini 2011, p. 138; German Ethics Council 2010, pp. 34–35; Gibbons 2009, p. 13). As expressed by Hansson and Bjorkman, “it should be in society’s interest to adjust the legislation so that strong promises of secrecy can be both given and upheld” (2006, p. 292). There are also several examples of practical implementation of this strategy. For example, the Estonian Human Genes Research Act specifically indicates that data and samples contained in the Estonian Genebank cannot be accessed by the police (note, however, that this law does not apply to other biobanks operating in the country) (The Parliament of Estonia 2000), and Hofmann reports that in Norway, the “Supreme Court […] decided that biological material gained for medical purposes could not be used for forensic purposes” (2006, p. 131). The Certificates of Confidentiality issued by the National Institutes of Health in the USA (Kaye 2006, pp. 16–17) are supposed to allow researchers to stop the police from accessing research collections, but their legal status and effectiveness have been the subject of debate (Gunn and Joiner 2009; Beskow et al. 2008).

To be fully effective, however, this policy must be implemented on a very high level in the legal system, such as in a special law, as is the case in Estonia. Otherwise, if forensic access in a particular case is deemed to be necessary to protect some important societal interests (as may be the case in investigations of serious crimes), these interests can sometimes legally prevail over interests in privacy and confidentiality (Rothstein and Talbott 2006; Laurie et al. 2013).

## The third solution: limit and disclose it

If relevant types of forensic access cannot be fully excluded in a given legal system (either due to the nature of the system or to a lack of political will), the system can be designed in a way that allows such access only in the most exceptional circumstances. Perhaps the greatest threat to public trust is constituted by a situation where forensic access becomes a routine procedure rather than an exception (Kaye 2006, p. 25). Limiting relevant types of forensic access goes some way toward protecting donor privacy, and public trust in research (hence ensuring participation). A wide variety of legal instruments, from confidentiality and data protection laws to such documents as the Certificates of Confidentiality issued by NIH, can be effective, even if in some circumstances defeasible, means to limit forensic access.

We believe that if some type of forensic access to research collections is likely to be perceived by the subjects as a reason not to donate their biological materials, there are good ethical reasons to make this type of access impossible, or at least severely restricted. If, however, relevant types of forensic access cannot be fully ruled out by legislative means, the possibility of forensic use should be communicated to the donors. As stated by Hansson and Bjorkman, “It is common to promise that only the researchers conducting the particular study will have access to the data. If this promise cannot be legally upheld, it will have to be adjusted accordingly” (2006, p. 292).

This practice of disclosing potential forensic access is, to the best of our knowledge, extremely rare. The UK Biobank took a lead by stating in its donor information leaflet (but not in the consent form) that if forced by courts, it would grant access to the police to the individual’s information, samples or test results (UK Biobank 2010, p. 9). Generation Scotland (a sister institution of UK Biobank) followed suit. A similar recommendation can be found in the report produced by the Scottish government on collection and storage of Guthrie cards (Laurie, Hunter and Cunningham-Burley 2013). The majority of other collections still do not explicitly mention the possibility of forensic use in their consent forms or informational materials.

This strategy of disclosure has several advantages. First, relevant considerations are honored and donors are not deceived. Second, it is easy to implement—a simple sentence in a consent form can be enough. Third, advance disclosure removes the potential of unexpected surfacing of the information on forensic access to diminish public trust. One may worry, however, that disclosure may reduce the number of donors. Of course, the extent of this risk is an empirical question. It can be said, though, that if relevant types of forensic access are regulated in a way that makes them rare exceptions rather than a norm, perhaps, if properly communicated to the donors, this will not result in significant decrease of participation.

How should the disclosure be implemented? In the current situation, adding one general sentence to the research consent form would perhaps be enough. A good example would be the phrase “Access to the resource by the police or other law enforcement agencies will be acceded to only under court order, and [biobank] will resist such access vigorously in all circumstances,” taken from a document describing the UK Biobank Ethics and Governance Framework (UK Biobank 2007). Assuming that the sentence is accurate, and research participants have an opportunity to ask for clarifications, this should be sufficient.

It may be argued, however, that at least for some donors the possibility of some types of forensic access (for instance, investigations of crimes against the donor or donor’s family members, or postmortem paternity testing) may constitute a reason to donate their materials for long-time storage rather than a reason to abstain from donating. This is fully compatible with our main claim that *if* there are reasons to believe that *some* types of forensic access are likely to be relevant considerations against participation, *then* the possibility of *these* types of forensic access should be disclosed. If there are reasons for treating some types of forensic access as reasons to participate and other types of forensic access as reasons not to participate, this calls for a more fine-grained approach to consent. Still, *prima facie* it seems that it is more important to disclose reasons *against* participation than to disclose reasons *in favor* of participation, for only in the first case do we risk wrongful deception.

## Conclusions

The extant empirical studies on public attitudes toward forensic access indicate that at least some types of forensic access can be considered by a portion of the public to be relevant considerations. That is, availability of information on such types of forensic access may be important or even crucial in their judgments on whether to donate their identifiable materials to research biobanks. The question that arises is whether, if such types of forensic access are a real possibility, this information should be made available to the donors. This article suggests that the ethically preferable strategy is to make relevant types of forensic access legally impossible. If this cannot be achieved, the next best option is to limit relevant forensic access as much as possible. To the extent that relevant types of forensic access cannot be completely excluded by legal means, information on the potential of such forensic access should be communicated to the donors. This solution allows one to navigate the dilemma formulated at the beginning of the paper. On the one hand, it protects the individual interests by allowing the donors to make an informed choice and not be deceived. On the other hand, it protects the trust in biobanks by making relevant types of forensic access impossible, or at least very difficult.

This argument depends on a number of empirical premises that require further study and constitute fertile grounds for further research. It would be important to know which types of forensic access are likely to be relevant considerations. Therefore public attitudes should be assessed in relation to these different types of forensic access rather than to forensic access as a single construct. We also call for more transparency in regard to forensic access to non-forensic collections. It would be very helpful to know how often (and what types of) forensic access is attempted and how often it is granted. Empirical information of these sorts would be helpful in further shaping the argument.

## References

[CR1] Beskow Laura M, Dame Lauren, Jane Costello E (2008). Certificates of confidentiality and the compelled disclosure of research data. Science.

[CR2] Bexelius Christin, Hoeyer Klaus, Lynöe Niels (2007). Will forensic use of medical biobanks decrease public trust in healthcare services? Some empirical observations. Scandinavian Journal of Public Health.

[CR3] Bowman Diana M, Studdert David M (2011). Newborn screening cards: A legal quagmire. The Medical Journal of Australia.

[CR4] Bromwich Danielle, Millum Joseph (2013). Disclosure and consent to medical research participation. Journal of Moral Philosophy.

[CR5] Butler John M (2009). Fundamentals of forensic DNA typing.

[CR6] Cho Mildred K, Sankar Pamela (2004). Forensic genetics and ethical, legal and social implications beyond the clinic. Nature Genetics.

[CR7] Dyer C (2001). Use of confidential HIV data helps convict former prisoner. BMJ: British Medical Journal.

[CR8] Feinberg Joel (1984). Harm to self.

[CR9] German Ethics Council (2010). Human biobanks for research.

[CR10] Gibbons Susan MC (2009). Regulating biobanks: A twelve-point typological tool. Medical Law Review.

[CR11] Gunn Patrick P, Joiner Scott D (2009). Certificates should be strengthened. Science.

[CR12] Hansson Sven Ove, Bjorkman Barbro (2006). Bioethics in Sweden. Cambridge Quarterly of Healthcare Ethics.

[CR13] Hofmann Bjørn (2006). Forensic uses and misuses of DNA: A case report from Norway. Genomics, Society and Policy.

[CR14] Kaufman David J (2009). Public opinion about the importance of privacy in biobank research. The American Journal of Human Genetics.

[CR15] Kaye Jane (2006). Police collection and access to DNA samples. Genomics, Society and Policy.

[CR16] Laurie, Graeme, Kathryn Hunter and Sarah Cunningham-Burley. 2013. Storage, use and access to the scottish guthrie card collection: Ethical, legal and social issues. The Scottish Government. http://www.gov.scot/Resource/0044/00441799.pdf. Accessed 23 June 2015.

[CR17] Lee Steven B, Crouse Cecelia A, Kline Margaret C, Shewale Jaiprakash G, Liu Ray H (2013). Optimizing storage and handling of DNA extracts. Forensic DNA analysis: Current practices and emerging technologies.

[CR18] Lewis Celine (2013). Public views on the donation and use of human biological samples in biomedical research: A mixed methods study. BMJ Open.

[CR19] McCartney Carole (2004). Forensic DNA sampling and the England and Wales National DNA database: A skeptical approach. Critical Criminology.

[CR20] National Institute of Justice. 2012. Increasing the capacity of crime laboratories. http://www.nij.gov/topics/forensics/lab-operations/evidence-backlogs/Pages/increasing-capacity.aspx. Accessed 23 June 2015.

[CR21] Nuffield Council on Bioethics. 2007. Forensic uses of bionformation. Ethical issues.

[CR22] Rothstein Mark A, Talbott Meghan K (2006). The expanding use of DNA in law enforcement: What role for privacy?. The Journal of Law, Medicine & Ethics.

[CR23] Seiden Samuel C, Morin Karine (2002). The physician as gatekeeper to the use of genetic information in the criminal justice system. The Journal of Law, Medicine & Ethics.

[CR24] Tamburrini C, Lenk Ch (2011). What’s wrong with forensic uses of biobanks?. Biobanks and tissue research.

[CR25] The Federal Bureau of Investigation. 2015. CODIS—NDIS Statistics. https://www.fbi.gov/about-us/lab/biometric-analysis/codis/ndis-statistics. Accessed 23 June 2015.

[CR26] The Parliament of Estonia. 2000. Estonian Human Genes Research Act, 2000. https://www.riigiteataja.ee/en/eli/531102013003/consolide. Accessed 10 March 2015.

[CR27] UK Biobank. 2007. UK Biobank Ethics and Governance Framework. http://www.ukbiobank.ac.uk/wp-content/uploads/2011/05/EGF20082.pdf. Accessed 22 June 2015.

[CR28] UK Biobank 2010. Information Leaflet. http://www.ukbiobank.ac.uk/wp-content/uploads/2011/06/Participant_information_leaflet.pdf. Accessed 10 March 2015.

[CR29] Webster, Andrew et al. 2008. Public attitudes to third party access and benefit sharing: Their application to UK Biobank. Final report. Science and Technology Studies Unit (SATSU) University of York. http://egcukbiobank.org.uk/meetingsandreports. Accessed 10 March 2015.

